# Genomic Epidemiology of Carbapenemase Producing *Klebsiella pneumoniae* Strains at a Northern Portuguese Hospital Enables the Detection of a Misidentified *Klebsiella variicola* KPC-3 Producing Strain

**DOI:** 10.3390/microorganisms8121986

**Published:** 2020-12-13

**Authors:** João Perdigão, Cátia Caneiras, Rita Elias, Ana Modesto, Anton Spadar, Jody Phelan, Susana Campino, Taane G. Clark, Eliana Costa, Maria José Saavedra, Aida Duarte

**Affiliations:** 1 Research Institute for Medicines (iMed.ULisboa), Faculty of Pharmacy, Universidade de Lisboa, 1649-033 Lisboa, Portugal; jperdigao@ff.ulisboa.pt (J.P.); rita.elias@tecnico.ulisboa.pt (R.E.); ammodesto96@gmail.com (A.M.); 2 Laboratory of Microbiology Research in Environmental Health (EnviHealthMicro Lab), Institute of Environmental Health (ISAMB), Faculty of Medicine, Universidade de Lisboa, 1649-026 Lisboa, Portugal; ccaneiras@medicina.ulisboa.pt; 3 Institute of Preventive Medicine and Public Health (IMP&SP), Faculty of Medicine, Universidade de Lisboa, 1649-026 Lisboa, Portugal; 4 Department of Microbiology and Immunology, Faculty of Pharmacy, Universidade de Lisboa, 1649-033 Lisboa, Portugal; 5 Faculty of Infectious and Tropical Diseases, London School of Hygiene and Tropical Medicine, London WC1E 7HT, UK; anton.spadar@lshtm.ac.uk (A.S.); jody.phelan@lshtm.ac.uk (J.P.); Susana.campino@lshtm.ac.uk (S.C.); taane.clark@lshtm.ac.uk (T.G.C.); 6 Faculty of Epidemiology and Population Health, London School of Hygiene and Tropical Medicine, London WC1E 7HT, UK; 7 Serviço de Patologia Clínica, Centro Hospitalar de Trás-os-Montes e Alto Douro, 5000-508 Vila Real, Portugal; eliana.santos.costa@gmail.com; 8 Laboratory Medical Microbiology, Department of Veterinary Sciences, CITAB-Centre for the Research and Technology Agro-Environmental and Biological Sciences, University of Trás-os-Montes and Alto Douro, 5000-801 Vila Real, Portugal; saavedra@utad.pt; 9 Centro de Investigação Interdisciplinar Egas Moniz, Instituto Universitário Egas Moniz, 2829-511 Monte da Caparica, Portugal

**Keywords:** *Klebsiella pneumoniae*, *Klebsiella variicola*, KPC-3, OXA-48, Gram-negative, molecular epidemiology, carbapenemase, whole-genome sequencing, *Enterobacteriaceae*, Portugal

## Abstract

The evolutionary epidemiology, resistome, virulome and mobilome of thirty-one multidrug resistant *Klebsiella pneumoniae* clinical isolates from the northern Vila Real region of Portugal were characterized using whole-genome sequencing and bioinformatic analysis. The genomic population structure was dominated by two main sequence types (STs): ST147 (*n* = 17; 54.8%) and ST15 (*n* = 6; 19.4%) comprising four distinct genomic clusters. Two main carbapenemase coding genes were detected (*bla*_KPC-3_ and *bla*_OXA-48_) along with additional extended-spectrum β-lactamase coding loci (*bla*_CTX-M-15_, *bla*_SHV-12_, *bla*_SHV-27_, and *bla*_SHV-187_). Moreover, whole genome sequencing enabled the identification of one *Klebsiella variicola* KPC-3 producer isolate previously misidentified as *K. pneumoniae*, which in addition to the *bla*_KPC-3_ carbapenemase gene, bore the chromosomal broad spectrum β-lactamase *bla*_LEN-2_ coding gene, *oqxAB* and *fosA* resistance loci. The *bla*_KPC-3_ genes were located in a *Tn4401b* transposon *(K. variicola* *n* = 1; *K. pneumoniae* *n* = 2) and *Tn4401d* isoform (*K. pneumoniae* *n* = 28). Overall, our work describes the first report of a *bla*_KPC-3_ producing *K. variicola,* as well as the detection of this species during infection control measures in surveillance cultures from infected patients. It also highlights the importance of additional control measures to overcome the clonal dissemination of carbapenemase producing clones.

## 1. Introduction

*Klebsiella pneumoniae* (*Kp*) has emerged as an increasingly resistant pathogen and has shown an ability to express several intrinsic and acquired mechanisms making the species multi-resistant to clinical antimicrobial classes. Using sequencing and phylogenetic analysis, isolates in the *Kp* complex have been classified into seven phylo-groups (Kp1 to Kp7) [1,2]. The phenotypic and biochemical characteristics of the *Kp* complex are extremely similar under standard microbiological conditions, which makes it difficult to distinguish phylo-groups by biochemical tests and routine microbiological methods. The inaccurate identification of *Kp* complex phylo-groups is leaving gaps in knowledge and has clinical implications within health care systems. *K. variicola* (*Kv*) isolates are included in phylogenetic group Kp3 and are becoming increasingly significant from a clinical standpoint and associated with increased mortality [3,4]. Of great concern is that *Kp* and *Kv* can co-exist in the same host. Garza-Ramos et al. identified two isolates from an infected Mexican patient: one susceptible *Kv* isolate and a second carbapenemase-producing *Kp* isolate [5]. Usually, *Kv* isolates display low antibiotic resistance rates; however, this pattern has changed over time due to an increase in reports of multidrug-resistant isolates, including extended-spectrum β-lactamase (ESBL) (*bla*_CTX-M-15_) or carbapenemase (*bla*_KPC-2_, *bla*_NDM-5_, *bla*_IMI-2_) producing isolates [6,7]. Moreover, reports of multidrug-resistant and hypervirulent *Kv* isolates have been increasing from other different sources, such as plants and environmental isolates, which highlights a key role of the environment as reservoirs of antimicrobial resistance genes and hypervirulent isolates [3,8].

Recently, it has been reported that *Kv* display a large spectrum of virulence factors, similar to *Kp,* encoded by genes located in chromosomal gene clusters or in genetic accessory elements. Additionally, it has been shown using genomic and phylogenetic analyses that these gene clusters and elements confer infective potential for community-acquired isolates [9,10,11]. The mechanisms underpinning the virulence of pathogenic bacteria as well as those determining antibiotic resistance are important and widely studied topics in clinical microbiology. However, due to the lack of a suitable identification approach for routine microbiological use and the potential impact of species of the *Klebsiella* complex on clinical outcomes and settings, the aim of this study was to analyze the whole-genome sequences of *Kp* isolates from a hospital in the northern region of Portugal to determine their relatedness and to detect possible high-risk clones, antimicrobial resistance markers and putative virulence factors.

## 2. Materials and Methods

### 2.1. Bacterial Isolates

Thirty-one isolates of *Kp*-producing carbapenemases, from 28 patients identified by Centro Hospitalar de Trás os Montes e Alto Douro (CHTMAD) in Vila Real, Portugal, were characterized using whole-genome sequencing and bioinformatic analysis. The biological samples collected were inoculated on specific culture medium CHROMagar mSuperCARBA media (bioMérieux, La Balme-les-Grotte, France) for detection of carbapenemases for rectal and nasal swabs, and additional Columbia agar with 5% sheep blood plates for clinical specimens (bioMérieux, La Balme-les-Grotte, France). Identification and antibiotic susceptibility testing were performed using the VITEK Microbial Detection System (bioMérieux, La Balme-les-Grotte, France). The European Committee on Antimicrobial Susceptibility Testing (EUCAST) guidelines were used for interpretation of antimicrobial susceptibility testing (v.8) [12]. Phenotypic KPC carbapenemase detection was performed with the double-disc synergy test with boronic acid inhibition [13].

### 2.2. Whole-Genome Sequencing

DNA for whole genome sequencing were extracted from cultures grown overnight in Mueller–Hinton agar, using the NZY Tissue gDNA Isolation kit (NZYTech, Lisboa, Portugal), as per the manufacturer’s recommendations. Sequencing libraries were prepared using Covaris and the New England Biolabs (NEB) NEBNext Ultra DNA Library Prep Kit (NEB, Ipswich, MA, USA E7370) following the manufacturer’s recommended protocol and sequenced using an Illumina HiSeq 4K platform with paired-end 2 × 151 bp reads. De novo assembly was performed using the Unicycler assembly pipeline (https://github.com/rrwick/Unicycler) with SPAdes (v.3.8) software [14]. All assemblies were carried out with a k-mer length of 127 bp and contig N50 ranged between 147.5 and 504.6 Kbp, contig count between 54 and 297 Kbp, and the longest contig between 156.4 and 1046.0 Kbp. Genomic sequences were deposited on the European Nucleotide Archive under Study PRJEB41591.

### 2.3. Phylogenetic Analysis

Phylogenetic reconstruction from high-quality SNPs obtained by mapping raw sequencing reads to the reference genome of *Kp* IA65 (GenBank Accession NZ_CP030070.1). The reference genome was selected using refRank (https://gitlab.com/s.fuchs/refRank) from a total of 289 *Kp* genomes available at GenBank with an assembly level of “complete”. Trimmomatic (v0.36) software was used for read trimming and quality control, where reads <36 bp length and with an average PHRED score below 20 in 4 bp sliding windows were excluded. Surviving reads were mapped to the reference genome using the Burrows–Wheeler Aligner tool (BWA-MEM algorihm) [15,16]. BAM deduplication and read realignment around indels were done using Picard Tools and GATK v.3.6 [17]. Variant calling was performed using both SAMtools/BCFtools and GATK (UnifiedGenotyper) software and only concordant variants were retained for further downstream analysis. The following filtering steps were performed to obtain a set of high-quality SNPs: (i) coverage-validation, a missing call assigned if coverage depth did not reach a minimum of 20 reads or if none of the nucleotides accounted for 75% of the total coverage; (ii) only reference genome positions yielding 49/50 bp unique k-mers were retained in order to remove SNP positions associated with low mappability; (iii) sites displaying an excess of 10% missed calls were removed; (iv) SNP sites within 10 bp were removed (pruning) [18]. A total of 64,106 high quality SNPs were identified across the 31 isolates included in the study and concatenated into pseudo DNA molecules for phylogenetic inference. The Generalized Time Reversible model allowing for a proportion of invariable sites was selected upon fitting different nucleotide substitution models to this dataset (R/Phangorn package;—log likelihood: 426,121.7703; AIC weight: 0.49881). A maximum-likelihood phylogenetic tree was constructed using PhyML as implemented in Seaview and tree topology statistically assessed using the approximate Likelihood Ratio Test (aLRT) [19,20]. The Interactive Tree of Life tool (iTOL, https://itol.embl.de/) [21] was used to annotate the tree and a minimum spanning tree was also constructed using Phyloviz (http://online2.phyloviz.net/users/login) and the goeBURST algorithm therein implemented using the pairwise comparison analysis method [22]. Genomic clusters were defined as a group of two or more isolates within 23 SNPs [23].

### 2.4. In Silico MLST, Plasmid Replicons, Drug Resistance Associated Genes and Capsular Types

In silico Multilocus Sequence Typing MLST was carried out using MLST 1.8 (https://cge.cbs.dtu.dk/). Abricate software was used for plasmid replicon detection using the PlasmidFinder database (https://cge.cbs.dtu.dk/), with the following cut-off values: minimum of 60% coverage and 95% identity (80% for Col-like plasmids) [24]. Antimicrobial resistance genes were screened using the AMRFinder software (https://ncbi.nlm.nih.gov/pathogens/antimicrobial-resistance/AMRFinder/) along with the NCBI Bacterial Antimicrobial Resistance Reference Gene Database (Accession PRJNA313047) using a 60% coverage and 95% identity thresholds. The genetic context of *bla*_CTX-M-15_, *bla*_KPC-3_ and *bla*_OXA-48_ was investigated and compared across different isolates by aligning the respective contigs using the MAFFT 7 online server [25]. Reconstruction of the genetic context was performed using BLAST (blastn) across full length contigs, or on the region comprising up to 10 Kbp upstream and downstream of the resistance gene of interest, against the GenBank nucleotide collection. *Tn4401* isoform identification was performed using BLAST and sequence alignment against *Tn4401b* and *Tn4401a* (GenBank Accessions KT378598 and EU16011, respectively). Capsular type (K-*locus*) and O-locus types were predicted using Kaptive Web (https://github.com/kelwyres/Kaptive-Web). SpeciesFinder 2.0 was used to confirm/validate initial phenotypic-based identification [26].

## 3. Results

### 3.1. Whole Genome Sequencing Enables Identification of K. variicola Misidentified as K. pneumoniae

The study comprises a total of 31 contemporary *Klebsiella* spp. isolates recovered in a Northern Central Hospital. All isolates were initially identified as *Kp* based on phenotypical biochemical testing (VITEK) and were subjected to WGS with the objective of gaining further insight on the evolutionary epidemiology, resistome, mobilome and virulome of these *Klebsiella* complex species. Upon whole genome sequencing and in silico species confirmation, all but one isolate was identified as *Kp* (*n* = 30). The remaining isolate was identified as *Kv* (*n* = 1) with high confidence by SpeciesFinder (v2.0). Eighteen of the 31 isolates were obtained from six clinical specimens: urine (*n* = 10), blood (*n* = 2), respiratory tract secretions by double-lumen catheter (RTS/DLC) (*n* = 2); catheter (*n* = 2); biopsy (*n* = 1) and intra-operative swab (IOSwab; *n* = 1) (Table 1). According to control measures established by Infection Prevention and Control Commission of the Hospital, 13 out of the 31 *Kp* isolates were recovered from rectal swabs from four colonized patients (P3, P8, P20, P27) upon admittance to the hospital and from eight colonized patients staying on the wards: Medicine (P1, P12, P28); intensive care unit (ICU) (P2, P26); Surgical (P6, P16) and Nephrology (P7). In addition, patient 28 was also colonized with Kv5163. Five patients attending the emergency were infected with *Kp* isolates obtained from urine (P4, P14, P17), blood (P15) and sputum (P20), which suggests colonization/infection outside the hospital.

### 3.2. Genomic Population Structure and High-Resolution Phylogenomic Analysis Unveils Multiple Healthcare-Associated Transmission Clusters

To ascertain the genomic population structure, in silico MLST identification was carried out for each isolate in order to assign them to different sequence types (ST). Among the 30 *Kp*, eight STs were detected: ST147 (*n* = 17 isolates, 16 patients), ST15 (*n* = 7, 6 patients), ST34 (*n* = 1), ST1029 (*n* = 1), ST29 (*n* = 1), ST46 (*n* = 1), ST307 (*n* = 1), and ST280 (*n* = 1). Each of the P5 and P20 patients had two isolates assigned to the ST147 and ST15, respectively. Moreover, patient P28, admitted to the Medicine ward, was colonized with *Kv* ST4197 and after two weeks a *Kp* ST280 strain was identified.

**Figure 1:**
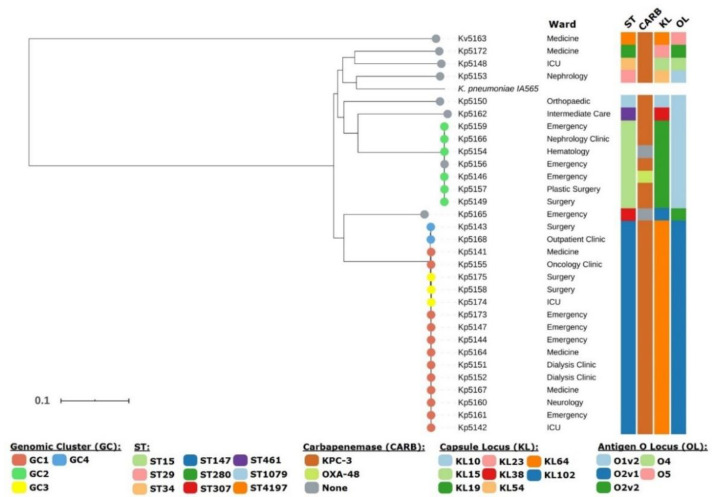
Genome-wide phylogenetic tree of 30 *K. pneumoniae* (*Kp*) isolates and one *K. variicola* (*Kv*) isolate analyzed in the present study. The phylogeny depicted is based on 64,106 high quality SNPs obtained from mapping raw sequence reads against the reference strain *Kp* IA65 also present in the tree. The tree is topologically structured by sequence type (ST) and is annotated with the ward, ST, capsular locus type (KL), and antigen O locus type (OL) according to the legend. Colored tree tips highlight the different genomic clusters (GCs) to which the isolate has been assigned or if it is non-clustered (light grey).

**Figure 2:**
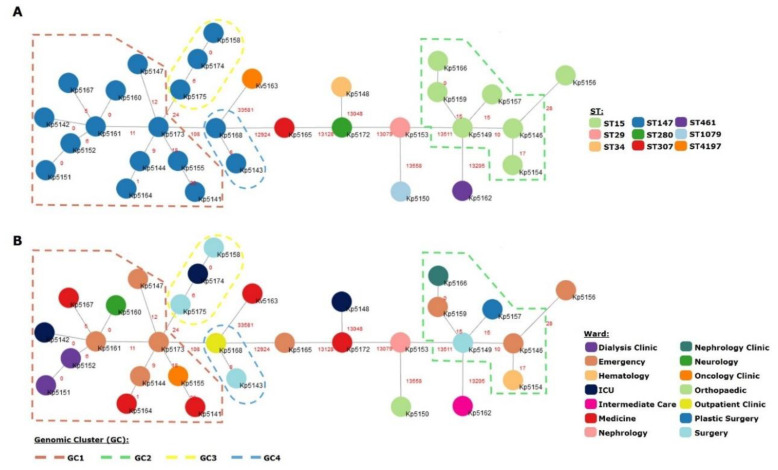
Minimum spanning trees (MST) constructed using the goeBURST algorithm with the pairwise comparison method for all isolates included in the study. The MSTs are shown annotated with the genomic clusters and node coloring reflect the ST (**A**) or ward from which the isolate was obtained (**B**). Four different GCs (GC1-4) were detected and highlighted on the tree reflecting ongoing transmission across the wards from the studied hospital.

High-resolution phylogenomic analysis based on 64,106 high quality SNPs enabled the construction of a phylogenetic tree whose topological structure was congruent with the MLST-based classification with *Kv*5163 phylogenetically placed farther apart from all *Kp* isolates included in the study (Figure 1). Both ST147 and ST15 strains formed monophyletic clades and no association with a specific ward was identified. Interestingly, *Kp*5156 and *Kp*5159, both isolated from P20, did not form a monophyletic clade and were separated by 35 SNPs, suggesting different infection/colonization events with a ST15 strain (Figure 2). Contrarily, *Kp*5151 and *Kp*5152, did form a monophyletic clade and were indistinguishable through the present phylogenetic scenario (distance: 0 SNPs). Using a 23 SNP cutoff to delineate clusters enabled the identification of four genomic clusters (GC1-4) with the two largest clusters, GC1 (*n* = 12) and GC2 (*n* = 6), being comprised by ST147 and ST15 isolates, respectively (Figure 2). Both GC1 and GC2 show a high dispersal through different wards of the studied hospital but were more prevalent from patients attending the Emergency as 4/12 and 3/6 isolates from GC1 and GC2 were in fact isolated from patients in this ward (Figure 1).

The two other GCs are shown to involve ST147 strains (GC3 and GC4). Noteworthily, GC3 isolates are in fact separated from GC1 isolates by 24 SNPs, one SNP above the threshold for cluster assignment and likely highlights a group of ST147 strains derived from GC1, unsampled cases or missed links in the transmission chains that may take the form of undetected colonized patients. The fact that GC4 isolates form a monophyletic clade nested within the GC1 branch of the tree lends further support to this hypothesis (Figure 1).

### 3.3. KPC-3 as the Main Driver of Carbapenem Resistance in Northern Portugal and First Description of KPC-3 Producing K. variicola

According to antimicrobial susceptibility (Supplementary Table S1) and regarding the resistance genes found in *Kp*, we identified two carbapenemase-coding genes (*bla*_KPC-3_ and *bla*_OXA-48_), four genes coding for extended-spectrum β-lactamases (ESBLs), namely *bla*_CTX-M-15_, *bla*_SHV-12_, *bla*_SHV-27_, *bla*_SHV-187_), and eight genes coding for narrow/broad spectrum beta-lactamases (*bla*_TEM-1_, *bla*_TEM-33_, *bla*_SHV-1_, *bla*_SHV-11_, *bla*_SHV-26_, *bla*_SHV-28_, *bla*_OXA-1_, *bla*_OXA-9_). The *aac, aad* and *aph* aminoglycoside-modifying enzyme genes were detected in most (27/31; 87.1%) of the isolates, except in three *Kp* and one *Kv.* Trimethoprim–sulfamethoxazole resistance was encoded by *sul* and *dfr* genes. The *sul1* and *sul2* genes were detected in 13 isolates each, and one isolate had both genes. Moreover, the four isolates without aminoglycoside-modifying enzyme genes did not have *sul* and *dfr* genes. The *qnr, oqxAB* and *aac(6′)-lb-cr5* plasmid-mediated quinolone resistance (PMQR) genes and mutations in chromosomal *gyrA* or *parC* loci were detected in these isolates. The *fosA* gene, which codes for fosfomycin resistance, was identified in all isolates; chloramphenicol acetyltransferase genes were also identified: catA1 (*n* = 4), catA2 (*n* = 1), and catB3 (*n* = 4). The rifampicin adenosine diphosphate (ADP) ribosylating transferase arr−3 gene was detected in 10 isolates (Table 2). The *Kv*5163 had the chromosomal broad spectrum β-lactamase *bla*_LEN-2_, the *bla*_KPC-3_, *oqxAB* and *fosA* genes. 

Inspection of the genetic environments of *bla*_KPC-3_, *bla*_CTX-M-15_, and *bla*_OXA-48_ revealed twenty-five out of the 28 KPC-3 producing isolates had the same genetic environment as part of the *Tn4401d* isoform, which is characterized by a 68 bp deletion between *istB* and the *bla*_KPC_ gene when compared with the *Tn4401b* isoform [27]. This *Tn4401b* isoform was identified in the remaining three isolates *Kp*5148 (ST34), *Kp*5162 (ST451) and *Kv*5163. The co-existence of *Kv*5163 with *Kp*5172 in same patient is supportive of distinct dissemination events regarding the origin of the *bla*_KPC-3_ in these strains.

The *bla*_CTX-M-15_ gene was carried on an IS*Ecp1* insertion sequence (IS) element and was detected in six isolates belonging to ST15 (*n* = 3 isolates) and in each one of ST147, ST307, ST280. The *bla*_OXA-48_ gene, detected in *Kp*5146 (ST15) isolate, was inserted into an IncL/M-type plasmid, a replicon exclusively found in this strain, with 100% coverage and identity to reference replicon type under accession no. JN626286. Part of the plasmid (IncL/M p5146) was found in a contig where the region of *bla*_OXA-48_ gene was identical to pOXA48a [28], described as having part of *Tn1999* upstream and a *lysR* gene downstream encoding a regulatory protein. In addition, in this Kp5146 isolate, four replicons were found: IncFIB(K)Kpn3; IncFIB(Mar); IncFII(pMET) and ColRNAI_1. Using the criterion of >95% nucleotide identity for large plasmids and >80% for Col-like plasmids and >96% coverage with the reference replicon sequences [24], among the 31 isolates five major replication loci were detected, namely IncFIB(K) (*n* = 24, 77.4%), IncFIA(HI1) and IncFII-1-pKP91 (*n* = 18, 58.1%), IncN-1 (*n* = 17, 54.8%). Sixteen replicons were found among 31 isolates (Table 3). Eight (*n* = 8) out 16 *Kp* ST147 isolates had as part of IncN-1 plasmid a complex sul1-type that contain a partial duplication of the 3′-CS. Between both copies of the 3′-CS there is a variable region harbouring a *qnrB6* gene and, in the region between the 5′CS and the first 3′CS we detected the presence of the *aac(6′)-Ib-cr5*; *arr-3*; *dfr*A27 and *aad*A16 gene cassettes. The IncFIB group was the most abundantly distributed by all ST types (including Kv5163) represented by four subvariants (IncFIB (pQil)_1; IncFIB (K)_1_Kpn3; IncFIB (pKPHS1); IncFIB (Mar)_1_pNDM).

### 3.4. Virulome of K. pneumoniae Highlights the Occurrence of Multiple Virulence Factors and ST-Specific Patterns

Regarding the virulence genes found in *Kp* and *Kv* isolates, the distribution of capsular locus (KL) types denoted specific associations with ST, where each KL type occurred only in one ST type and was common to all isolates belonging to same ST type (Table 4). The exception was *Kv*5163 with KL64, similar to *Kp* ST147 isolates but with diverging antigen O locus (OL) types O5 and O2v1, respectively. The O1v2 types were dispersed throughout the *Kp* ST15, *Kp* ST1079, *Kp* ST29 and *Kp* ST461.

Both bacterial species *Kp* and *Kv* showed a profile of virulence-associated determinants, specifically fimbrial adhesins (*fimH*-1), and siderophore *entB* (enterobactin). The *iroE* and *iutA*-18 genes were found in all strains, however the other genes integrating the *iucABCD* and *iroBCDN* siderophore gene clusters were not detected. Further analysis on the genetic context of these two genes and its distribution across other *Enterobactereaceae* species revealed that these genes are likely co-localized to the chromosome and show a wide distribution: these *iroE* homologues were detected across the chromosomes of *K. pneumoniae*, *K. quasipneumoniae*, *K. variicola* and *K. quasivariicola* (phylogroups Kp1-4) [2]; the iutA homologues showed an even wider distribution that included the former species but also *Raoultella* spp, *Citrobacter* spp., *Kluyvera* spp., etc. with varying homology identities. The yersiniabactin siderophore cluster represented by *ybtQ* gene was identified in *Kp* ST147 and ST15 genomes (Table 4). However, the iron-uptake system siderophore (*kfu*) was present in *Kp* ST15, *Kp* ST461 and *Kv* ST4197 strains but not *Kp* ST147. The Type VI secretion system (T6SS) mediated type-1 fimbriae expression, cell adherence, invasion, and in vivo colonization, is represented by type VI membrane-targeting phospholipase effector Tle1 and Tli1 immunity proteins [29,30] and was detected in 19 isolates, the majority of which were ST147 strains (Table 4).

## 4. Discussion

Genomic sequences of thirty-one isolates initially identified as *Kp* were used for phylogenetic analyses and to investigate their genomic content for antimicrobial resistance genes, plasmid replicon types and virulence genes. Through whole genome sequencing we were able to correctly identify a KPC-3-producing *Kv* isolate previously misidentified as *Kp* during routine laboratory diagnosis. The present study thus highlights the presence of *Kp* and *Kv* isolates from the same patient at different time-points. The correct identification of the *Klebsiella* species can be technically challenging for most clinical microbiology laboratories since several species share a similar colony morphology and biochemical profile. A second potential case of colonization by *Kv* was detected in a patient in this same hospital setting, and also identified as *Kp*, but was excluded from the study due to mixed infection with another *Kp* strain (data not shown). This latter isolate yielded a larger genome assembly with duplicate resistance gene sequences mapping to distinct contigs that despite several attempts were not possible to isolate. The misidentified *Kv* isolate and the mixed colonization described stress the emerging importance of *Kv* as a host for drug resistance determinants and the existing technical challenges that may underestimate its epidemiological importance. The presence of *bla*_LEN-2_, a broad-spectrum b-lactamase that confers resistance to ampicillin is characteristic for *Kv* strains [31]. The *oqxAB*, *fosA*, *bla*_SHV_ or *bla*_LEN_ genes are ubiquitous and considered the ancestral resistome from the *Klebsiella* complex [32,33]. Carbapenemase-producing *Kv* isolates have been described in different countries, namely KPC-2 in USA, Europe and in Asia [3]. In Portugal the widespread distribution of KPC-3 among *Kp* isolates in different hospitals was associated to successful high-risk clones ST147 [34]; ST307; ST15 [27] and ST14 [35,36] located in a *Tn4401d* isoform. The genetic context of *bla*_KPC_ is known to have contributed greatly to the spread of KPC producers in many countries, and the emergence of non-CG258 KPC-*Kp* isolates in France was linked to dissemination of these clones from Portugal [37]. To our knowledge, we report the first KPC-3 producing *Kv* isolate with the *bla*_KPC-3_ gene mapped to the *Tn4401b* isoform, a less common genetic environment also found in two additional *Kp* isolates from this study (*Kp*5148 and *Kp*5162) which also suggests a potential scenario for cross-species lateralization of KPC-3.

Herein, the population structure found across these isolates was dominated by two of the high-risk clones mentioned above: ST147 (*n* = 17) and ST15 (*n* = 7). Both strains formed monophyletic clades comprising all transmission clusters found in the present study. The identification of these genomic clusters reflecting ongoing transmission was performed on the basis of a SNP distance threshold of 23 SNPs as previously reported [23]. In fact, 23/30 (76.7%) *Kp* isolates were within one of the four genomic clusters identified and therefore highlight the highly clonal nature of this population structure and its dissemination across multiple wards in the studied setting. This level of uncontrolled dissemination stresses the importance of additional infection control measures such as the rapid detection of colonization and hygiene/decontamination of possible contaminated environmental sources. Additionally, the ability to discriminate at the genome-wide level allowed for the discrimination within ST147 strains of two isolates that are here shown to be epidemiologically unrelated to the rest of the ST147 strains (distance: 108 SNPs) further stressing the low discriminatory power of MLST.

A diverse set of plasmids has been characterized in *Kp* and although these can vary in terms of length and incompatibility types, IncFIIK and IncFIBK are considered the most prevalent across this species [38]. In this study, these and other Inc. groups co-existed across different STs. Across ST147 (*n* = 17 isolates) strains a marked variation was observed for the number of replicons found per isolate (*n* = 2–6; 11.8% and 35.3%, respectively), whereas for ST15 (*n* = 7) strains the number of replicons per isolate was lower with three different replicon types per isolate as the predominant value (57%). In contrast, the Kv5163 isolate had five detectable replicons of which FIB(K)Kpn3 and ColRNAI _1 were the predominant replicons found for *Kp*, while from the three remaining replicons only IncFII(pMet) was exclusively detected in the *Kv* strain. Furthermore, for ST147 strains, the *bla*_KPC−3_ was colocalized to an IncN-1 plasmid whose repN showed 100% identity with plasmid R46 identified in Salmonella serovar Typhimurium (GenBank accession number AY046276). The same genetic background for *bla*_KPC-3_ was described in isolates identified on non-hospitalized patients (three different community laboratories) in Portugal [34]. The results obtained in this study also highlight its putative importance as a genetic platform for cross species mobilization of carbapenem resistance determinants. Moreover, also colocalized to this IncN-1 plasmid, a complex sul1-type Class1 integron was found, harboring in region 5′ CS five gene cassettes the *aac(6′)-Ib-cr5*; *arr-3*; *dfr*A27 and *aad*A16 and following the 2nd copy of 3′ CS of the sixth *qnrB*6 gene cassette. This latter structure appears to be absent from the KPC-3-bearing IncN plasmid detected in Salmonella serovar Typhimurium (accession number AY046276).

A different set of ST147 (*n* = 4) strains was found to include the *sul2* gene on a class 1 integon associated to *aac(6′)-Ib*, *aad*A1, *bla*_OXA-9_, *bla*_TEM-1_, *aph(6)-Id*, *aph(3”)-Ib* gene cassettes. Interestingly, this genetic profile was found for all ST15 isolates, although with three variants regarding aminoglycoside resistance gene cassettes *aac(6′)-Ib*, *aad*A1 (3 isolates), *aac(3)-IIa* (2 isolates) and *aad*A1 (2 isolates). The *sul2* has been previously described on small non-conjugative plasmids in association with the *aph(6)-Id*, *aph(3″)-Ib* gene although its presence has also been reported on large plasmids, some of which are transmissible and carrying several resistance determinants [39]. Although we could not phase the location of this gene with the replicon type, in this study *sul2* was detected in contigs as large as ≥30 Kbp.

*Kp* and *Kv* infections have limited therapeutic options due to extensive drug resistance and successful immune escape mechanisms of these pathogens, which exhibit a high number of virulence factors. The leading virulence factors are mostly associated with capsular serotype [40] and the LPS (O antigen) O1 and O2 serotypes are the most common underpinning 50–68% of all *Klebsiella* spp. infections [40]. Twenty-nine out of the 30 isolates included in the study had the O1 and O2 serotypes associated with variants v1 or v2 while *Kp*5148 (ST34) and *Kv*5163 belonged to the O4 and O5 serotypes, respectively. The O1 strains are generally more resistant to the bactericidal effect of human serum, which has been attributed to the d-galactan II component in O1 antigen [41] which is not present in the O4 and O5 strains and there are clinical data suggesting that these antigens play a role in virulence or pathogenicity. The high prevalence observed for O1/O2 serotypes across the studied isolates is paralleled by a recent study in a Lisbon hospital and is congruent with a selective advantage by these serotypes [27]. However, while the Lisbon study mentioned demonstrated that the ST147 strains from this study and the ones from Lisbon (both KPC-3 producers) exhibit the same KL and OL types, the ST15 strains herein characterized differ in respect to these serotypes. This observation may be associated with the fact that ST15 strains detected recently in Lisbon were ESBL producers but not carbapenemase producing strains and highlights the serotype switching event that is herein associated with the phylogenetic branch of carbapenemase producing ST15 strains in the northern region of Portugal.

Additionally, microbial adherence and invasion of host cells are critical steps in the infection process and comprise additional virulence determinants with most clinical *Kp* isolates expressing two types of fimbrial adhesins, type 1 and type 3 fimbriae. Twenty-eight *Kp* and the *Kv* isolates had the *fim* gene cluster coding for the type 1 fimbriae which mediates adhesion to mannose-containing structures. Moreover, all but two ST147 isolates (*Kp*5143 and *Kp*5155) were concomitantly positive for the *mrk* gene cluster (type 3 fimbriae). The type 3 fimbriae encoded by the *mrk* gene cluster is characterized by their ability to mediate adhesion to different structures, through MrkD adhesin, as well as role in biofilm formation, by MrkA adhesin [42].

The presence of multiple iron transport systems in *Kp* suggests that iron acquisition systems are needed during infection. Four iron transport systems, *entB*, *ybtS*, *iutA*, and *kfu* are usually present, although a single siderophore-dependent iron acquisition system may be sufficient for bacterial colonization. The siderophore enterobactin (Ent) are common to all *Kp* and conserved in the chromosome as core genes. Nevertheless, the enhanced virulence in the majority of *Kp* lineages associated with liver abscess carry virulence plasmid encoding *iuc* and *iro* genes inserted in specific siderophore systems, aerobactin (Iuc) and salmochelin (Iro) [43,44]. The *iucABCDiutA* siderophore gene cluster was first reported on pCoIV-K30 in *E. coli* and on pLVPK of *K. pneumoniae* CG43 [43]. The *iroBCDEN* gene cluster, first described in *Salmonella enterica*, and was also found either on the chromosome or a transmissible plasmid in the uropathogenic *Escherichia coli* [45] and in *Kp* four structures of the salmochelin (*iro*) locus were described, one of them without the *iroE* gene [44]. The allele *iutA-18* showed 100% coverage with 93.73–95% identity with the core chromosomal paralog of *iutA* encoding a *TonB*-dependent siderophore receptor. In addition, the *iroE* gene found herein is located in the chromosome and showed 100% coverage with 98.83–99.25% identity with an alpha/beta hydrolase. We believe that these are homologous genes acquired throughout the evolutionary history of the *Klebsiella* genus but unrelated to the *iucABCDiutA* and *iroBCDEN* gene clusters.

The iron uptake system (*kfu*) contributes to invasive disease and could play a part in the pathogenicity of bloodstream infections caused by *Kv* [46]. In this study, all *Kp* ST15 strains and the *Kv*5163 isolate carried the *kfu* gene, which was not present in *Kp* ST147 isolates. However, the ST147 strains and *Kv*5163 had the type VI secretion system (T6SS), which is widely implicated in microbial antagonism and mediates interactions with host eukaryotic cells and in the environment in competition with other species. This could explain of presence of this type VI secretion system in *Kv*5163 isolate.

## 5. Conclusions

The present study contributes with novel data on the genomic epidemiology, its resistome, mobilome and virulome of carbapenemase producing *K. pneumoniae* in a hospital in northern Portugal. To our knowledge this is the largest study involving carbapenemase producing isolates from Portugal carried out at the genome-wide level while simultaneously comprising the first report of a KPC-3 producing *K. variicola* worldwide with the *bla_KPC-3_* gene being located in the rather uncommon horizontal gene transfer element *Tn4401b* isoform. This work further highlights the technical challenges associated with *K. variicola* identification at the species level during routine laboratory diagnosis with WGS providing a correct identification and the importance for more strict control measures to gain further control on the highly clonal dissemination.

## Figures and Tables

**Table 1:** Characteristics of the 31 isolates recovered from 28 patients colonized/infected by *K. pneumoniae* and *K. variicola* isolates.

ID	Isolates	Patient	Sample	Hospital Ward	Date	ST
5141	*K. pneumoniae*	P1	Rectal swab	Medicine	2 May 2018	ST147
5142	*K. pneumoniae*	P2	Rectal swab	ICU	3 May 2018	ST147
5144	*K. pneumoniae*	P3	Rectal swab	Emergency	4 May 2018	ST147
5147	*K. pneumoniae*	P4	Urine	Emergency	6 May 2018	ST147
5151	*K. pneumoniae*	P5	Dialysis liquid	Dialysis Clinic	15 May 2018	ST147
5152	*K. pneumoniae*		Purulent exudate	Dialysis Clinic	15 May 2018	ST147
5143	*K. pneumoniae*	P6	Rectal swab	Surgery	16 May 2018	ST147
5160	*K. pneumoniae*	P7	Rectal swab	Neurology	25 May 2018	ST147
5161	*K. pneumoniae*	P8	Rectal swab	Emergency	26 May 2018	ST147
5155	*K. pneumoniae*	P9	Urine	Oncology Clinic	29 May 2018	ST147
5158	*K. pneumoniae*	P10	Urine	Surgery	4 June 2018	ST147
5164	*K. pneumoniae*	P11	Urine	Medicine	4 June 2018	ST147
5167	*K. pneumoniae*	P12	Rectal swab	Medicine	4 June 2018	ST147
5168	*K. pneumoniae*	P13	Urine	Outpatient Clinic	11 June 2018	ST147
5173	*K. pneumoniae*	P14	Urine	Emergency	17 June 2018	ST147
5174	*K. pneumoniae*	P15	Blood	Emergency	19 June 2018	ST147
5175	*K. pneumoniae*	P16	Rectal swab	Surgery	19 June 2018	ST147
5146	*K. pneumoniae*	P17	Urine	Emergency	3 May 2018	ST15
5149	*K. pneumoniae*	P18	Hepatic liquid	Surgery	9 May 2018	ST15
5154	*K. pneumoniae*	P19	Blood	Hematology	28 May 2018	ST15
5159	*K. pneumoniae*	P20	Rectal swab	Emergency	25 May 2018	ST15
5156	*K. pneumoniae*		Sputum	Emergency	30 May 2018	ST15
5157	*K. pneumoniae*	P21	Biopsy	Plastic Surgery	4 June 2018	ST15
5166	*K. pneumoniae*	P22	Urina	Nephrology clinic	6 June 2018	ST15
5148	*K. pneumoniae*	P23	Urine	ICU	7 May 2018	ST34
5150	*K. pneumoniae*	P24	Urine	Orthopaedic	13 May 2018	ST1079
5153	*K. pneumoniae*	P25	RTS/DLC ^a^	Nephrology	21 May 2018	ST29
5162	*K. pneumoniae*	P26	Rectal swab	ICU	29 May 2018	ST461
5165	*K. pneumoniae*	P27	Rectal swab	Emergency	5 June 2018	ST307
5163	*K. variicola*	P28	Rectal swab	Medicine	29 May 2018	ST4197
5172	*K. pneumoniae*		Rectal swab	Medicine	18 June 2018	ST280

^a^ Respiratory tract secretions by double-lumen catheter.

**Table 2:** Resistance determinants of thirty-one multi-resistant *K. pneumoniae* and *K. variicola* isolates.

Isolates ID	Sequence Type	Genomic Cluster	*bla*_Carb	*bla*_ESBL	*bla*_narrow Spectrum			Aminoglycoside		aac(6′)-Ib-cr5	qnr	Phe	Rif	Fos	oqxAB	tmp	qacEdelta1	sul	tet
*K*p5141	ST147	GC1	KPC-3	CTX-M-15	SHV-11	TEM-1	OXA-1	*aph(6)-Id; aph(3″)-Ib*	*aac(3)-IIa*	*aac(6′)-Ib-cr5*		*cat*B3		*fosA*	*oqxAB*	*dfr*A14		*sul2*	
*K*p5142	ST147	GC1	KPC-3		SHV-11				*aad*A1					*fosA*	*oqxAB*	*dfr*A1	*qacEdelta1*	*sul1*	
*K*p5144	ST147	GC1	KPC-3		SHV-11				*aad*A16	*aac(6′)-Ib-cr5*	*qnr*B6	*-*	*arr-3*	*fosA*	*oqxAB*	*dfr*A27		*sul1*	
*K*p5147	ST147	GC1	KPC-3	-	SHV-11				*aad*A16	*aac(6′)-Ib-cr5*		*-*	*arr-3*	*fosA*	*oqxAB*	*dfr*A27	*qacEdelta1*	*sul1*	
*Kp* 5151	ST147	GC1	KPC-3	-	SHV-11				*aad*A16	*aac(6′)-Ib-cr5*	*qnr*B6	*-*	*arr-3*	*fosA*	*oqxAB*	*dfr*A27	*qacEdelta1*	*sul1*	
*Kp*5152	ST147	GC1	KPC-3	-	SHV-11				*aad*A16	*aac(6′)-Ib-cr5*	*qnr*B6	*-*	*arr-3*	*fosA*	*oqxAB*	*dfr*A27	*qacEdelta1*	*sul1*	
*Kp*5143	ST147	GC4	KPC-3		SHV-12	TEM-1		*aph(6)-Id; aph(3″)-Ib*			*qnrB1*			*fosA*	*oqxAB*	*dfr*A14	*qacEdelta1*	*sul2*	*tet(A)*
*Kp*5160	ST147	GC1	KPC-3	-	SHV-11				*aad*A1			*-*	*-*	*fosA*	*oqxAB*	*dfrA1*	*qacEdelta1*	*sul1*	
*Kp*5161	ST147	GC1	KPC-3	-	SHV-11				*aad*A1			*-*	*-*	*fosA*	*oqxAB*	*dfrA1*	*qacEdelta1*	*sul1*	
*Kp*5155	ST147	GC1	KPC-3		SHV-11	TEM-33			*-*			*-*	*-*	*fosA*	*oqxAB*	*-*			
*Kp*5158	ST147	GC1	KPC-3	-	SHV-1				*aad*A16	*aac(6′)-Ib-cr5*	*qnrB6*	*-*	*arr-3*	*fosA*	*oqxAB*	*dfr*A27	*qacEdelta1*	*sul1*	
*Kp*5164	ST147	GC1	KPC-3	-	SHV-11				*aad*A16	*aac(6′)-Ib-cr5*	*qnrB6*	*-*	*arr-3*	*fosA*	*oqxAB*	*dfr*A27	*qacEdelta1*	*sul1*	
*Kp*5167	ST147	GC1	KPC-3	-	SHV-11				*aad*A1			*-*	*-*	*fosA*	*oqxAB*	*dfrA1*	*qacEdelta1*	*sul1*	
*Kp*5168	ST147	GC1	KPC-3		SHV-12	TEM-1	OXA-9	*aph(6)-Id; aph(3″)-Ib*	*aac(6′)-Ib; aadA1*		*qnr*B1	*-*	*-*	*fosA*	*oqxAB*	*dfr*A14		*sul2*	*tet(A)*
*Kp*5173	ST147	GC1	KPC-3	-	SHV-11				*aad*A16	*aac(6′)-Ib-cr5*	*qnr*B6	*-*	*arr-3*	*fosA*	*oqxAB*	*dfr*A27	*qacEdelta1*	*sul1*	
*Kp*5174	ST147	GC3	KPC-3	-	SHV-1				*aad*A16	*aac(6′)-Ib-cr5*	*qnr*B6	*-*	*arr-3*	*fosA*	*oqxAB*	*dfr*A27	*qacEdelta1*	*sul1*	
*Kp*5175	ST147	GC3	KPC-3	-	SHV-11				*aad*A16	*aac(6′)-Ib-cr5*	*qnr*B6	*-*	*arr-3*	*fosA*	*oqxAB*	*dfr*A27	*qacEdelta1*	*sul1*	
*Kp*5146	ST15	GC2	OXA-48	CTX-M-15	SHV-28	TEM-1	OXA-1	*aph(6)-Id; aph(3″)-Ib*	*aac(3)-IIa*	*aac(6′)-Ib-cr5*	*qnr*B1	*cat*A1*; cat*B3	*-*	*fosA*	*oqxAB*	*dfr*A14		*sul2*	
*Kp*5149	ST15	GC2	KPC-3	-	SHV-28	TEM-1	OXA-9	*aph(6)-Id; aph(3″)-Ib*	*aac(6′)-Ib; aadA1*			*-*	*-*	*fosA*	*oqxAB*	*dfr*A14		*sul2*	
*Kp*5154	ST15	GC2		CTX-M-15	SHV-28	TEM-1		*aph(6)-Id; aph(3″)-Ib*	*aac(3)-IIa*		*qnr*B1	*cat*A1*; cat*B3	*-*	*fosA*	*oqxAB*	*dfr*A14		*sul2*	
*Kp*5159	ST15	GC2	KPC-3	-	SHV-28	TEM-1	OXA-9	*aph(6)-Id; aph(3″)-Ib*	*aadA1*		*qnr*B1	*cat*A1	*-*	*fosA*	*oqxAB*	*dfr*A14		*sul2*	*tet(A)*
*Kp*5156	ST15		KPC-3	CTX-M-15		TEM-1	OXA-9	*aph(6)-Id; aph(3″)-Ib*	*aac(6′)-Ib; aadA1*		*qnr*B1	*-*	*-*	*fosA*	*oqxAB*	*dfr*A14		*sul2*	
*Kp*5157	ST15	GC2	KPC-3	-	SHV-28	TEM-1	OXA-9	*aph(6)-Id; aph(3″)-Ib*	*aac(6′)-Ib; aadA1*			*-*	*-*	*fosA*	*oqxAB*	*dfr*A14		*sul2*	
*Kp*5166	ST15	GC2	KPC-3	-	SHV-28	TEM-1	OXA-9	*aph(6)-Id; aph(3″)-Ib*	*aad*A1		*qnr*B1	*cat*A1	*-*	*fosA*	*oqxAB*	*dfr*A14		*sul2*	*tet(A)*
*Kp*5148	ST34		KPC-3	-	SHV-26				*-*			*-*	*-*	*fosA*	*oqxA10; oqxB19*	*-*			
*Kp*5150	ST1079		KPC-3	-	SHV-1	TEM-1	OXA-9	*aph(6)-Id; aph(3″)-Ib*	*aac(6′)-Ib; aadA1*			*-*	*-*	*fosA*	*oqxA; oqxB19*	*dfr*A14		*sul2*	
*Kp*5153	ST29		KPC-3	-	SHV-187	TEM-1	OXA-9	*aph(6)-Id; aph(3″)-Ib*	*aac(6′)-Ib; aadA1*		*qnrS1*	*-*	*-*	*fosA*	*oqxA; oqxB25*	*dfr*A14		*sul2*	
*Kp*5162	ST451		KPC-3	-	SHV-1				*-*			*-*	*-*	*fosA*	*oqxA10; oqxB25*	*-*			
*Kp*5165	ST307			CTX-M-15	SHV-28	TEM-1		*aph(6)-Id; aph(3″)-Ib*	*aac(3)-IIa; aad*A16	*aac(6′)-Ib-cr5*	*qnr*B6	*cat*A2	*arr-3*	*fosA*	*oqxA; oqxB19*	*dfr*A27	*qacEdelta1*	*sul1;sul2*	*tet(D)*
*Kv*5163	ST4197		KPC-3	-		LEN-2			*-*			*-*	*-*	*fosA*	*oqxA; oqxB15*	*-*			
*Kp*5172	ST280		KPC-3	CTX-M-15	SHV-27	TEM-1	OXA-1; OXA-9	*aph(6)-Id; aph(3″)-Ib*	*aac(3)-IIa; aac(6′)-Ib; aad*A1	*aac(6′)-Ib-cr5*	*qnr*B1	*cat*B3	*-*	*fosA*	*oqxA; oqxB19*	*dfr*A14		*sul2*	*tet(A)*

**Table 3:** Distribution of plasmid replicons identified among 31 whole genome sequenced *K.pneumoniae* and *K. variicola* isolates by PlasmidFinder database.

	*K. pneumoniae*								*K. variicola*		
Replicon	ST147 **	ST15	ST461	ST1079	ST29	ST307	ST280	ST34	ST4197	Identity/%	N° Isolates/%
IncFIA (HI1)	6	7	1	1	1	1	1			94.59–100	18/58.1
IncFIB (pQil)_1_pQil			1 *						1 *	100	02/06.4
IncFIB (K)_1_Kpn3	12 (9 *)	7 *	1	1		1 *	1		1 *	98.93–100	24/77.4
IncFIB (pKPHS1)	14									97.32–100	14/45.5
IncFIB (Mar)_1_pNDM	1				1				1 *	99.77–100	03/09.7
IncFII_1_pKP91	10	5	1	1				1		85.47–88.55	18/58.1
IncFII (Yp)	6	5		1	1		1			85.71	14/45.5
IncFII (pMET)_1_pMET1									1	98.09	01/03,2
IncR_1	4 (2 *)	2 *				1				99.2–100	07/22.5
IncN_1	16							1		99.42	17/54.8
IncL/M (pOXA-48)		1 *								100	01/03.2
IncX4_1		1								99.73	01/03.2
repA-pKPC-2								1		99.71	01/03.2
Col (MG828)1	1									93.51	01/03.2
ColRNAI_1	4 (2 *)	6	1	1				1	1	85.5–100	14/45.5
ColpVC_1	1									98.45	01/03.2

* The number followed by * represents the isolates with 100% nucleotide coverage and the identity to reference replicon type. ** number of isolates for STs: ST147-17 isolates; ST15-7 isolates; the remaining 1 isolate each has ST type.

**Table 4:** Distribution of virulent determinants against thirty-one multiresistant *K. pneumoniae* and *K. variicola* isolates.

Isolates ID	Sequence Type	*wzi*	K_locus	O_locus	Enterobactin *	Ferric Iron **	Ybt ***	Type I Fimbriae	Type 3 Fimbriae	Type VI SSE
*Kp*5141	ST147	*wzi*64	KL64	O2v1	*entB*		*ybtQ*	*fimH*	*mrkD*_12	*tle*1*; tli*1
*Kp*5142	ST147	*wzi*64	KL64	O2v1	*entB*		*ybtQ*	*fimH*	*mrkD*_12	*tle*1*; tli*1
*Kp*5144	ST147	*wzi*64	KL64	O2v1	*entB*		*ybtQ*	*fimH*	*mrkD*_12	*tle*1*; tli*1
*Kp*5147	ST147	*wzi*64	KL64	O2v1	*entB*		*ybtQ*	*fimH*	*mrkD*_12	*tle*1*; tli*1
*Kp*5151	ST147	*wzi*64	KL64	O2v1	*entB*		*ybtQ*	*fimH*	*mrkD*_12	*tle*1*; tli*1
*Kp*5152	ST147	*wzi*64	KL64	O2v1	*entB*		*ybtQ*	*fimH*	*mrkD*_12	*tle*1*; tli*1
*Kp*5143	ST147	*wzi*64	KL64	O2v1	*entB*					
*Kp*5160	ST147	*wzi*64	KL64	O2v1	*entB*		*ybtQ*	*fimH*	*mrkD*_12	*tle*1*; tli*1
*Kp*5161	ST147	*wzi*64	KL64	O2v1	*entB*		*ybtQ*	*fimH*	*mrkD*_12	*tle*1*; tli*1
*Kp*5155	ST147	*wzi*64	KL64	O2v1	*entB*					
*Kp*5158	ST147	*wzi*64	KL64	O2v1	*entB*		*ybtQ*	*fimH*	*mrkD*_12	*tle*1*; tli*1
*Kp*5164	ST147	*wzi*64	KL64	O2v1	*entB*		*ybtQ*	*fimH*	*mrkD*_12	*tle*1*; tli*1
*Kp*5167	ST147	*wzi*64	KL64	O2v1	*entB*		*ybtQ*	*fimH*	*mrkD*_12	*tle*1*; tli*1
*Kp*5168	ST147	*wzi*64	KL64	O2v1	*entB*			*fimH*	*mrkD*_12	*tle*1*; tli*1
*Kp*5173	ST147	*wzi*64	KL64	O2v1	*entB*		*ybtQ*	*fimH*	*mrkD*_12	*tle*1*; tli*1
*Kp*5174	ST147	*wzi*64	KL64	O2v1	*entB*		*ybtQ*	*fimH*	*mrkD*_12	*tle*1*; tli*1
*Kp*5175	ST147	*wzi*64	KL64	O2v1	*entB*		*ybtQ*	*fimH*	*mrkD*_12	*tle*1*; tli*1
*Kp*5146	ST15	*wzi*19	KL19	O1v2	*entB*	*kfuA*_3	*ybtQ*	*fimH*		
*Kp*5149	ST15	*wzi*19	KL19	O1v2	*entB*	*kfuA*_3	*ybtQ*	*fimH*		
*Kp*5154	ST15	*wzi*19	KL19	O1v2	*entB*	*kfuA*_3	*ybtQ*	*fimH*		
*Kp*5159	ST15	*wzi*19	KL19	O1v2	*entB*	*kfuA*_3	*ybtQ*	*fimH*		
*Kp*5156	ST15	*wzi*19	KL19	O1v2	*entB*	*kfuA*_3		*fimH*		
*Kp*5157	ST15	*wzi*19	KL19	O1v2	*entB*	*kfuA*_3		*fimH*		
*Kp*5166	ST15	*wzi*19	KL19	O1v2	*entB*	*kfuA*_3	*ybtQ*	*fimH*		
*Kp*5148	ST34	*wzi*50	KL15	O4	*entB*			*fimH*		*tle*1*; tli*1
*Kp*5150	ST1079	*wzi*236	KL10	O1v2	*entB*			*fimH*		
*Kp*5153	ST29	*wzi*115	KL54	O1v2	*entB*			*fimH*		
*Kp*5162	ST451	*wzi*38	KL38	O1v2	*entB*	*kfuA*_26		*fimH*		
*Kp*5165	ST307	*wzi*173	KL102	O2v2	*entB*			*fimH*		*tle*1*; tli*1
*Kv*5163	ST4197	-	KL64	O5	*entB*	*kfuA*_8		*fimH*		*tle*1*; tli*1
*Kp*5172	ST280	*wzi*82	KL23	O2v2	*entB*			*fimH*		*tle*1*; tli*1

* *entB* gene represents the Enterobactin gene cluster (*entA; entB; entC; entD; entE; entF; fepA; fepB; fepC; fepD; fepG; fes; ybdA*); ** *kfuA* gene represents the *kfuABC* operon; *** *ybtQ* gene represents the Yersiniabactin gene cluster (*fyuA; irp1; irp2; ybtA; ybtE; ybtP; ybtQ; ybtS; ybtT; ybtU; ybtX*).

## Supplementary Materials

The following are available online at https://www.mdpi.com/2076-2607/8/12/1986/s1, Table S1: Antimicrobial susceptibility of 30 *K. pneumoniae* isolates and one *K. variicola* isolate identified from 28 patients.
