# Supplementary material for: Genomic Epidemiology of Carbapenemase Producing Klebsiella pneumoniae Strains at a Northern Portuguese Hospital Enables the Detection of a Misidentified Klebsiella variicola KPC-3 Producing Strain
Source: Microorganisms. 2020 Dec 13;8(12):1986. doi: 10.3390/microorganisms8121986 (PMC7763156; doi:10.3390/microorganisms8121986)
Supplement: Supplementary file 1 [file microorganisms-08-01986-s001.pdf]

Table S1. Antimicrobial susceptibility of 30 *K. pneumoniae* isolates and one *K. variicola* isolate identified from 28 patients*

ID	Isolate	MLST	Patient	Cefoxitin	Cefotaxime	Ceftazidime	Gentamicin	Imipenem	Ciprofloxacin	sxt **
5141	*K. pneumoniae*	ST147	P1	R (10)	R (06)	R (06)	R (15)	R (11)	R (15)	R (06)
5142	*K. pneumoniae*	ST147	P2	R (08)	R (06)	R (06)	R (15)	R (10)	R (06)	R (06)
5144	*K. pneumoniae*	ST147	P3	R (11)	R (11)	R (06)	R (15)	R (15)	R (10)	R (06)
5147	*K. pneumoniae*	ST147	P4	R (11)	R (06)	R (06)	R (15)	R (11)	R (10)	R (06)
5151	*K. pneumoniae*	ST147	P5	R (11)	R (06)	R (06)	R (15)	R (11)	R (08)	R (06)
5152	*K. pneumoniae*	ST147		R (06)	R (08)	R (06)	R (15)	R (08)	R (06)	R (06)
5143	*K. pneumoniae*	ST147	P6	R (06)	R (08)	R (06)	R (15)	R (08)	R (06)	R (06)
5160	*K. pneumoniae*	ST147	P7	R (11)	R (11)	R (06)	R (15)	R (10)	R (06)	S (18)
5161	*K. pneumoniae*	ST147	P8	R (06)	R (06)	R (06)	R (15)	R (06)	R (06)	R (06)
5155	*K. pneumoniae*	ST147	P9	R (08)	R (08)	R (06)	S (18)	R (10)	R (10)	R (06)
5158	*K. pneumoniae*	ST147	P10	R (11)	R (06)	R (06)	S (18)	R (15)	R (14)	R (06)
5164	*K. pneumoniae*	ST147	P11	R (11)	R (06)	R (06)	S (18)	R (15)	R (06)	R (06)
5167	*K. pneumoniae*	ST147	P12	R (08)	R (06)	R (06)	S (18)	R (15)	R (08)	R (06)
5168	*K. pneumoniae*	ST147	P13	R (11)	R (06)	R (06)	R (10)	R (15)	R (06)	R (06)
5173	*K. pneumoniae*	ST147	P14	R (10)	R (06)	R (06)	R (15)	R (10)	R (06)	R (06)
5174	*K. pneumoniae*	ST147	P15	R (10)	R (06)	R (06)	S (18)	R (15)	R (06)	R (06)
5175	*K. pneumoniae*	ST147	P16	R (10)	R (06)	R (06)	R (11)	R (11)	R (06)	R (06)
5146	*K. pneumoniae*	ST15	P17	R (11)	R (06)	R (06)	R (06)	R (15)	R (06)	R (06)
5149	*K. pneumoniae*	ST15	P18	R (11)	R (06)	R (06)	R (06)	R (11)	R (06)	R (06)
5154	*K. pneumoniae*	ST15	P19	R (18)	R (06)	R (06)	R (06)	S (23)	R (06)	R (06)
5159	*K. pneumoniae*	ST15	P20	R (11)	R (11)	R (06)	R (08)	R (12)	R (06)	R (06)
5156	*K. pneumoniae*	ST15		R (10)	R (10)	R (06)	R (08)	R (10)	R (06)	R (06)
5157	*K. pneumoniae*	ST15	P21	R (08)	R (10)	R (06)	S (18)	R (15)	R (06)	R (06)

5166	*K. pneumoniae*	ST15	P22	R (06)	R (06)	R (06)	R (15)	R (08)	R (06)	R (06)
5148	*K. pneumoniae*	ST34	P23	R (06)	R (06)	R (06)	S (20)	R (06)	R (20)	S (20)
5150	*K. pneumoniae*	ST1079	P24	R (14)	R (06)	R (06)	R (10)	R (12)	R (21)	R (06)
5153	*K. pneumoniae*	ST29	P25	R (12)	R (12)	R (06)	R (11)	R (11)	R (06)	R (06)
5162	*K. pneumoniae*	ST461	P26	R (18)	R (14)	R (08)	R (15)	I (18)	R (21)	S (26)
5165	*K. pneumoniae*	ST307	P27	R (12)	R (06)	R (06)	R (06)	S (22)	R (06)	R (06)
5163	*K. variicola*	ST4197	P28	R (18)	R (15)	R (08)	R (16)	R (15)	R (21)	S (23)
5172	*K. pneumoniae*	ST280		R (18)	R (06)	R (08)	R (06)	R (16)	R (15)	R (06)

* According to EUCAST Clinical Breakpoint Tables v. 08.0, valid from 2018-01-01

** sxt: Trimethoprim-sulfamethoxazole

R/I/S R - Resistant; I - Susceptible, increased exposure; S - Susceptible
